# In Vitro Analysis of the Antioxidant and Antiviral Activity of Embelin against Herpes Simplex Virus-1

**DOI:** 10.3390/microorganisms9020434

**Published:** 2021-02-19

**Authors:** Tony Elias, Lee H. Lee, Miriam Rossi, Francesco Caruso, Sandra D. Adams

**Affiliations:** 1 Department of Biology, Montclair State University, Montclair, NJ 07043, USA; tonyelias222@gmail.com (T.E.); leel@montclair.edu (L.H.L.); 2 Department of Chemistry, Vassar College, Poughkeepsie, NY 12604, USA; rossi@vassar.edu (M.R.); caruso@vassar.edu (F.C.)

**Keywords:** embelin, HSV-1, antiviral, antioxidant

## Abstract

Herpes simplex virus-1 (HSV-1) causes a wide range of infections from mild to life-threatening in the human population. There are effective treatments for HSV-1 infections that are limited due HSV-1 latency and development of resistance to current therapeutics. The goal of this study was to investigate the antioxidant and antiviral effects of embelin on HSV-1 in cultured Vero cells. Oxidative stress was verified by an extensive production of a reactive oxygen species (ROS) H_2_O_2_. Vero cells were infected with a recombinant strain of HSV-1 and antiviral assays, time course attachment, penetration, and post penetration assays, confocal microscopy, qPCR, and antioxidant assays were conducted. Our results lead to the conclusion that embelin is noncytotoxic at concentrations tested ranging from 20 to 70 µM. Treatment of HSV-1 virions with embelin resulted in 98.7–100% inhibition and affected the early stage of HSV-1 infection of Vero cells, by inhibiting the attachment and penetration of HSV-1 virions to host cells. Treatment of virions with concentrations of embelin ranging from 35 to 60 µM significantly reduced the production of H_2_O_2._ In conclusion, embelin reduces oxidative damage caused by HSV-1 infection and is an effective antiviral to reduce the infection of HSV-1 in cultured Vero cells. Further studies are needed to explore the possibility of embelin as a medicinal agent.

## 1. Introduction

Herpes simplex virus-1 (HSV-1), a member of the family Herpesviridae, subfamily Alphaherpesvirinae, is an enveloped virus with a double-stranded (ds) DNA genome that causes a wide range of infections in humans from mild, uncomplicated mucocutaneous infections to life-threatening ones [1]. The dsDNA is enclosed within an icosahedral protein surrounded by a lipid bilayer [2]. HSV-1 has become a global health concern with 67% of the world population (estimated 3.4 billion) between the ages of 0 and 49 infected [3,4].

Entry of the virus to initiate lytic infection involves multiple surface glycoproteins [glycoprotein B (gB), glycoprotein C (gC), glycoprotein D (gD), glycoprotein H (gH), and glycoprotein L (gL)] interacting with surface receptors on the host cell. Specifically, gC and gB bind loosely to heparan sulfate proteoglycans followed by high-affinity binding of gD binding to entry receptors [5,6,7]. A number of cell receptors interact with gD. Some of these known receptors include the herpes virus entry mediator, nectin-1, and 3-*O*-sulfated heparan sulfate [8,9]. This induces a conformational change leading to the formation of a fusion complex composed of gB, gH, and gL. The viral nucleocapsid and tegument are released into the cytoplasm [8]. There is evidence for an atypical method of endocytic entry not mediated by clathrin-coated pits or caveolae [10]. Following entry of the virus into the cytoplasm, the virus particles travel to the nucleus where the viral DNA enters through the nuclear pore complex, and the viral genome replicates and is transcribed [11]. Virions are assembled in the cytoplasm and released by lysing the infected cell. Upon release, these new virions are capable of infecting new host cells [12].

HSV-1 is the viral causative agent of genital and oral herpes. These contagious and long-lasting infections cause painful blisters or ulcers at the site of infection [13]. The virus enters a latent phase in sensory ganglia, preventing clearance by the immune system [2]. 

Acyclovir, a nucleoside analog, its derivatives and their respective prodrugs, valacyclovir and famciclovir, are effective treatments for HSV-1 infections [4,14]. Acyclovir triphosphate is a substrate for the viral DNA polymerase that terminates viral DNA synthesis [15]. The latent stage and the development of resistance present limitations to the use of these drugs [8,16]. Additionally, despite efforts through the years, there does not exist a successful vaccine [17,18]. Since the virus can remain latent, it can be reactivated at any time. It is important to continue to search for novel antiviral compounds due to the ability of the virus to develop resistance to therapeutics. Research has reported that many biological plant-based compounds have demonstrated antiviral activity against HSV-1 infections. Plant based compounds such as curcumin, resveratrol, pomegranate rind extract, *Cornus canadensis* extract, and extracts from *Camellia sinensis* (epigallocatechin gallate and its lipophilic modifications, and theaflavins) have demonstrated activity against HSV-1 infections [19,20,21,22,23,24,25,26,27,28,29]. Several studies have determined that polyphenols inhibit adsorption and penetration during the viral lytic cycle [23,25,26,27,28,29].

Induction of oxidative stress is a mechanism used by several viruses. Human immunodeficiency virus (HIV) infection results in the accumulation of reactive oxygen species (ROS) mediated by glycoprotein 120 [30] and Tat proteins [31]. Influenza A viruses induce oxidative stress mediated by an over-production of ROS [32,33]. This increase in ROS was accompanied by a reduction in the antioxidant glutathione [34] and an increase in virus titer [32]. Surface antigens of hepatitis B virus (HBV) were reported to mediate ROS production; increased endoplasmic stress was linked to higher ROS production [35,36]. Curcumin, a polyphenol, inhibited an enzymatic reaction of apurinic/apyrimidinic endonuclease 1 (APE1). Inhibition of APE1 redox function decreased cell proliferation [37,38]. Curcumin inhibited Kaposi’s sarcoma-associated herpesvirus infection and inhibited angiogenesis [38]. Treatment with direct acting antivirals was found to improve the circulating redox status of patients with chronic hepatitis C infections [39].

Embelin (2,5-dihydroxy-3-undecyl-1,4-benzoquinone) is an orange solid derived from berries of the *Embelia ribes* plant found throughout India and is not water soluble; however, it can dissolve in organic solvents. Embelin has shown excellent antioxidant activity when scavenging the superoxide radical [40].

Embelin has demonstrated many biological activities including anxiolytic, anti-inflammatory, antioxidant, anticonvulsant, antidepressant, anthelmintic, antimicrobial, and anticancer [41,42,43,44,45,46,47,48]. Embelin has been studied as an antibacterial agent and was shown to be effective against *Staphylococcus aureus, Streptococcus pyogenes, Shigella flexneri, Shigella sonnei and Pseudomonas aeruginosa* [47]. A more recent study has shown that embelin provides promising antibacterial activity against some Gram-positive bacteria and bacteriostatic activity against Gram-negative ones [49]. Only minimal studies have been conducted to determine its antiviral properties. Extracts of *Embelia schimperi* (embelin and 5-*O*-methylembelin) inhibited infection of hepatitis C virus [50]. 

Embelin was found to have antiviral activity against influenza virus A/Puerto Rico/8/34 (H1N1) strain when applied directly to extracellular virions. In silico molecular docking analysis indicated that embelin interacts with the receptor binding domain of the viral hemagglutinin of influenza virus A [51]. Cell culture experiments combined with in silico docking analyses determined that embelin inhibited hepatitis B virus (HBV) by interacting with the HBV polymerase [52]. A recent computational study demonstrated the feasibility of covalent bond formation between embelin and the main protease of SARS-CoV-2, 3CL^pro^ [53].

Studies indicate that embelin is non-cytotoxic at concentrations tested [41,42,52]. Embelin was also shown to protect against oxidative stress in cells by reduction of its quinone ring and its long alkyl C10 tail may facilitate cell membrane insertion. The long lipophilic tail likely influences its biological activity in a highly specific manner. [41]. Due to its bioactivity and safe administration, the antioxidant embelin is an ideal compound to examine its antiviral activity, its effect on ROS production, and potential as a novel therapeutic agent to inhibit HSV-1 infections. The goal of this study is to investigate the antiviral effects of the antioxidant embelin on HSV-1 in cultured Vero cells.

## 2. Materials and Methods

### 2.1. Cell Culture

Vero cells (CCL-81) [American Type Culture Collection (ATCC), Manassas, VA, USA] were cultured until 70–80% confluent in Dulbecco’s Modified Eagle Medium (DMEM) supplemented with 5% fetal bovine serum (FBS) (Serum Source International, Charlotte, NC, USA) and 1% gentamicin sulfate (10 mg/mL, BioWhittaker, Lonza, VWR South Plainfield, NJ, USA) at 37 °C and 5% CO_2_. 

### 2.2. Preparation of Virus

A recombinant strain of HSV-1, GHSV-UL46 (ATCC VR-1544) which contains the sequence for green fluorescent protein (GFP) fused to the structural tegument protein pUL46 [54] was used for all experiments. Virus was passaged in T25 flasks and Vero cells were allowed to display full cytopathic effect (CPE) (MOI = 0.3). The media containing virus was then collected, centrifuged, and the supernatant stored at −80 °C.

### 2.3. Embelin

Purified embelin (Indofine Chemical Co., Hillsborough, NJ, USA) was dissolved in dimethyl sulfoxide (DMSO) to prepare a 10 mM stock. The stock was diluted in media to prepare concentrations ranging from 10 µM to 70 µM and stored at 4 °C.

### 2.4. Cell Proliferation Assays

Vero cells were cultivated for 24 h in a 96-well plate until 70–80% confluent. Increasing concentrations of embelin ranging from 20 µM to 70 µM were added to cells. After 1 h of incubation, the solution was aspirated. Media was then added to the wells and cells were incubated at 37 °C under 5% CO_2_ for 56 h. For quantitative analyses, a CellTiter 96 AQueous Cell Proliferation Assay (Promega, Madison, WI, USA) was utilized. This assay measures cell viability by the reduction of MTS tetrazolium to formazan, where absorbance of 490 nm is related to cell proliferation. Following manufacturer’s instructions, 20 µL of the MTS solution were added to the 100 µL of media in each well and the plate was incubated at 37 °C and 5% CO_2_ for one hour. The absorbance was measured using Infinite PRO 200 microplate reader (Tecan Life Sciences US, Raleigh, NC USA). Cell proliferation for each treatment condition was then calculated using the following formula: % proliferation = ((Treated cells—Blank)/(Cells only—Blank)) × 100%. Mean and standard deviation (SD) of four replicates were calculated using the Microsoft Excel function (Microsoft Office, New York, NY, USA).

### 2.5. Antiviral Assays

#### 2.5.1. Cell Proliferation Assay 

To determine embelin’s antiviral effects on HSV-1, Vero cells were cultivated for 24 h in a 96-well plate until 70–80% confluent. The virus was treated with increasing concentrations of embelin up to 54 µM for 1 h. Cells were infected (MOI = 1) with embelin treated virus one-hour post infection, any unabsorbed virus-containing media was aspirated and replaced with DMEM. Cells were incubated at 37 °C and 5% CO_2_. After 48 h, the cell proliferation was measured using the CellTiter 96 Aqueous Cell Proliferation Assay (Promega, Madison, WI, USA) as described above.

#### 2.5.2. Cell Viability Assay

A Viral ToxGlo assay (Promega, Madison, WI, USA) was conducted to quantitatively measure the cellular ATP, representing cell viability, and to determine the cytopathic effects (CPE) of viral infection. To determine embelin’s antiviral effects on HSV-1, Vero cells were cultivated for 24 h in a 96-well plate until 70–80% confluent. The virus was treated with increasing concentrations of embelin up to 54 µM for 1 h. Cells were infected (MOI = 1) with embelin treated virus for 1 h; one-hour post infection (hpi), any unabsorbed virus-containing media was aspirated and replaced with DMEM. Cells were incubated at 37 °C and 5% CO_2_ for 48 h. Following manufacturer’s instructions, 100 µL of ToxGlo reagent was added to the cells and the plate was incubated for 15 min. Luminescence, RLU (relative luminescence units), was measured with an Infinite Pro 200 microplate reader (Tecan Life Sciences US, Raleigh, NC, USA). Cell viability, determined by the ToxGlo assay, was then plotted against HSV-1 treatment using the following equation: % Viability = ((Cells infected with treated HSV-1—Blank)/(Cells only—Blank)) × 100%. Mean and SD of four replicates were calculated using the Microsoft Excel function.

### 2.6. Quantitative Polymerase Chain Reaction (qPCR)

To prepare for qPCR, the cells were grown in a 6-well plate at 37 °C and 5% CO_2_ until 70–80% confluent. The cells were infected with HSV-1 treated with 54 μM of embelin for one hour (MOI = 0.6). Uninfected cells served as a negative control. Cells infected with untreated HSV-1served as positive control. After 1 h of infection the media was aspirated from the cells and fresh media was added. DNA was collected 12 hpi using DNeasy Blood & Tissue kit (QIAGEN USA, Germantown, MD, USA). The primers HSV-1 gD forward 5′-CAACCCTACAACCTGACCATC-3′ and HSV-1 gD reverse 5′-TTGTAGGAGCATTCGGTGTAC-3′ were used. qPCR was performed using Applied Biosystems™ SYBR™ Green PCR Master Mix on MicroAmp™ Optical 96-Well Reaction Plate (ThermoFisher Scientific, Waltham, MA, USA). A 10-fold serial dilution was carried out up to the 10^−5^ dilution in order to determine the standard curve equation and R^2^. This calculation was performed using the Microsoft Excel function.

### 2.7. Microscopic Observations

#### 2.7.1. Inverted Microscopic Observation

Vero cells were cultivated for 24 h in a six-well plate until 70–80% confluent. Increasing concentrations of embelin ranging from 10 µM to 70 µM were added to cells. After 1 h of incubation, the solution was aspirated. Media was then added to the cells and treated cells were incubated at 37 °C under 5% CO_2_ and observed for a period of three days after treatment. Cells were examined, using an inverted microscope to observe morphological changes in the cells.

Vero cells were cultivated for 24 h in a six-well plate until 70–80% confluent HSV-1 virions were treated with increasing concentrations of embelin up to 54 µM for 1 h. Cells were infected (MOI = 1) with untreated or embelin treated virus for 1 h. One-hour post infection (hpi), any unabsorbed virus-containing media was aspirated and replaced with DMEM. Cells were incubated for 72 h at 37 °C and 5% CO_2_.

#### 2.7.2. Fluorescent Microscopy

To prepare cells for microscopy, the cells were grown on glass cover slips in 6-well plates. Vero cells were incubated at 37 °C and 5% CO_2_ until 70–80% confluent. When confluent, the cells were infected with treated or untreated HSV-1. The virus was treated with 54 μM of embelin for one hour. Uninfected cells served as a negative control. Cells infected with untreated virus served as positive control. After one hour of infection the media was aspirated from the cells and replaced with media. The cells on the coverslip were prepared and fixed 12 hpi. The cells were first treated with 300 μL of DAPI (4,6-diamidino-2-phenylindole) (Sigma Aldrich) stain for 5 min at 37 °C in the dark. Then the cells were fixed using 2–4% paraformaldehyde for 10–20 min followed by PBS wash. Mounting medium, containing 90% glycerol, and 10% phosphate buffered saline (PBS) was used to fix the coverslips on the slides. The slides were observed using a Zeiss Axio Scope A1 microscope (Zeiss US, Peabody, MA, USA).

### 2.8. Study of the Inhibitory Mechanism of Embelin on HSV-1 Infective Cycle

#### 2.8.1. Binding Assay

Vero cells were grown for 24 h in a 96-well plate until 70–80% confluent. The plate was pre-incubated at 4 °C for 30 min. Subsequently, the media was removed from the cells. The virus was treated with increasing concentrations (0, 20, 40, 54, and 60 µM) of embelin. Treated and untreated HSV-1 virions were incubated at room temperature for one hour followed by infection of the cells on ice. The plate was incubated for 1 h at 4 °C, then unbound HSV-1 was carefully aspirated from the cells using a multichannel pipettor. Fresh media was added to cells in each well [55]. The plate was incubated at 37 °C and 5% CO_2_ for 48 h and assayed using the Viral ToxGlo assay (Promega, Madison, WI, USA) as described previously. Mean and SD (four replicates) were calculated using the Microsoft Excel function.

#### 2.8.2. Penetration and Post Penetration Assay

Vero cells were grown in a 96-well plate until 70–80% confluent. The media was then removed from the cells and the cells were infected with 100 µL of HSV-1. The plate was incubated at 4 °C for 2 h to ensure attachment but not penetration of the HSV-1. At room temperature various concentrations (0, 20, 40, 50, 54, and 56 µM) of embelin were added. The plate was incubated at room temperature for 10 min for the penetration assay. For the post penetration assay, various concentrations of embelin (0, 20, 40, 50, 54, and 56 µM) were added after the virus particles entered the cells for 30 min at room temperature [55]. This was followed by the addition of 100 μL of 1X phosphate buffer saline (PBS) (pH 3.0) to each well to inactivate the virions. The media was removed carefully using a multichannel pipettor followed by the addition of 100 μL of fresh 5% FBS DMEM. Finally, after 48 h, viability was determined using the Viral ToxGlo (Promega, Madison, WI, USA) assay. The plate was read using the Infinite 2000 PRO microplate reader (Tecan Life Sciences US, Raleigh, NC, USA). Mean and SD (four replicates) were calculated using the Microsoft Excel function.

### 2.9. Study of Antioxidant and Antiviral Activity

The ROS-Glo H_2_O_2_ assay kit (Promega Corp., Madison, WI, USA) was used to assess oxidative stress induced by viral infection by measuring the levels of H_2_O_2_, a product of various reactive oxygen species (ROS). According to the manufacturer, an increase in H_2_O_2_ can reflect a general increase in cellular ROS levels. The ROS-Glo™ Assay uses a substrate that directly reacts with H_2_O_2_ to generate a luminescent signal proportional to the level of H_2_O_2_ present.

Vero cells were plated in a 96-well plate and allowed to reach 80% confluency, then infected with embelin treated or untreated virus for 1 h as described in the previous infection assays. The lytic assay protocol for product Cat#G8820 (Promega, Madison, WI, USA) was followed. Cells were then incubated at 37 °C in a humidified CO_2_ atmosphere for 18 h to allow infection, after which 20 µL of H_2_O_2_ substrate solution was added to each well and cells were incubated for an additional 6 h, for a total of 24 h of infection. After incubation, 100 μL of the ROS-Glo detection solution reaction was added to each well and incubated for 20 min. Luminescence, RLU (relative luminescence units) was measured with an Infinite Pro 200 microplate reader (Tecan Life Sciences US, Raleigh, NC, USA). 

### 2.10. Statistical Analyses

Student’s *t*-test statistical difference was performed using the Microsoft Excel function TTEST (Microsoft Office, New York, NY, USA). The analysis used two tailed distribution with two-sample unequal variance test. Cell viability for each treatment condition was then calculated using the following formula: % Viability = ((Treated cells − Blank)/(Cells only − Blank)) × 100%. Then, percent inhibition was calculated from the formula = 100 × (1 − (Embelin treatment − cells only)/(cells only − no treatment)); the MAX function was set at 100. Standard deviation (σ) was calculated using the excel function STDEV. The Z prime score was calculated using the formula: 1 − (3 × σ Positive control + 3 × σ negative control)/(Positive control − negative control) to assess the viability of the assay. A minimum of three replicates was performed in each of the conditions in question. Elimination of any dataset point outliers was done using the Data Rejection analysis by Dixon’s Q test.

## 3. Results

### 3.1. Cytotoxicity Study of Treatment of Vero cells with Embelin

**Figure 1:**
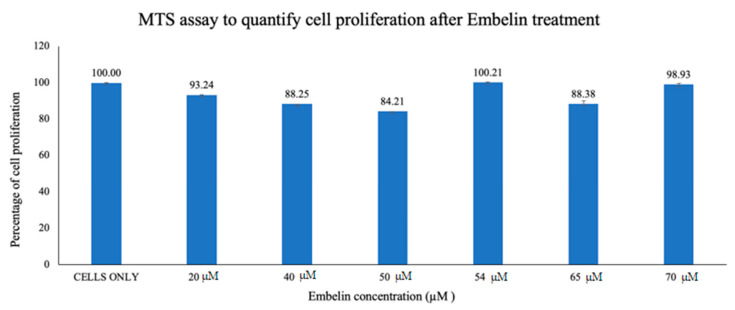
Vero cells were treated for 1 h with increasing concentrations of embelin. Embelin is non-cytotoxic to Vero cells at concentrations ranging from 20 to 70 µM. The results of cell proliferation assay of increasing concentrations of embelin treatment, 56 h post treatment are reported. Means are shown with standard deviations of four replicates.

Vero cells were treated with increasing concentrations of embelin, up to 70 µM concentration and 2% DMSO [27] although the maximum percentage of DMSO in this assay was 0.7%. The cell proliferation was quantified using the MTS assay. The cells were grown in a 96-well plate and incubated for 1 h with increasing concentrations of embelin (20–70 µM). The assay was performed after 56 h of incubation and absorbance was measured at 490 nm. Percent proliferation ranged from a low of 84.21%–100% (Figure 1). This demonstrates that embelin does not exert toxic effects on cells up to 70 µM concentrations.

### 3.2. Antiviral Effects of Embelin on HSV-1

#### 3.2.1. Proliferation Assay of Vero Cells Infected with HSV-1 and Embelin Treated HSV-1

**Figure 2:**
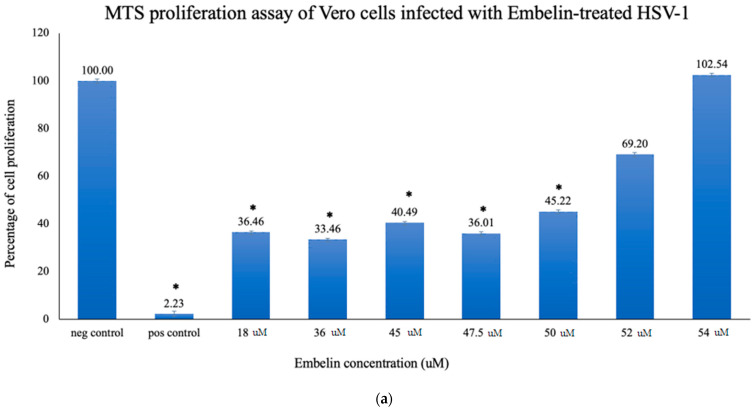

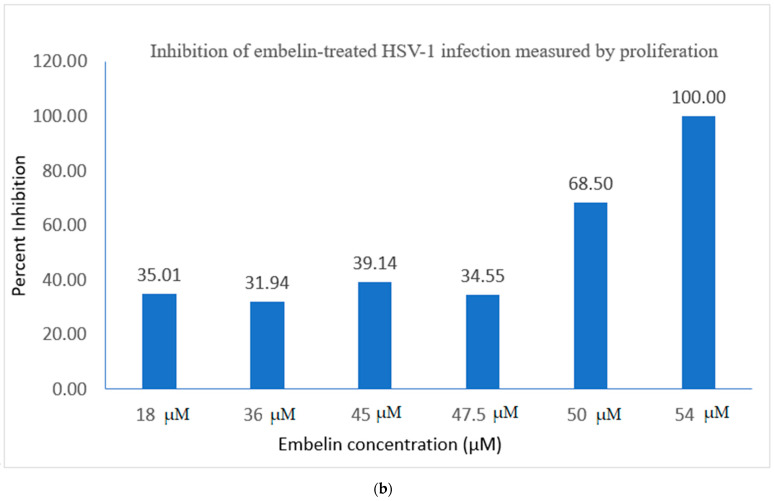
Treatment of HSV-1 virions affects cell proliferation of HSV-1 infected cells. (**a**) Cell proliferation determined by CellTiter 96 AQueous cell proliferation assay was utilized 48 hpi; * = significant difference *p* < 0.05 compared to the negative control. Means are shown with SD (four replicates). (**b**) The percent of antiviral inhibition of treatment of HSV-1 with different concentrations of embelin indicates that 54 µM concentration effectively inhibits HSV-1 infection.

To quantitatively study the effect of treatment of embelin on HSV-1 infection of Vero cells, proliferation of cells was assessed by the MTS assay. Concentrations of embelin at 0, 18, 36, 45, 47.5, 50, and 54 μM were used in this study. The results shown in Figure 2a demonstrated dose dependent inhibition with cell proliferation increasing with increasing concentration of embelin. The results indicate that treatment of HSV-1 with 54 μM embelin resulted in the highest cell proliferation (Figure 2a); this result is comparable to the proliferation of uninfected Vero cells. 

Percent inhibition following infection with embelin-treated HSV-1 was determined by the MTS assay. The calculated percentage of inhibition as measured by the MTS assay ranged from 35% when HSV-1 was treated with 18 µM embelin to 100%, when HSV-1 was treated with 54 µM concentration of embelin. The percent inhibition reached 100% inhibition with the 54 μM sample and 68% with the 50 μM treatment, respectively (Figure 2b). The percent of antiviral inhibition of embelin concentrations on HSV-1 with the greatest inhibition occurring with 54 μM treatment.

#### 3.2.2. Viral ToxGlo ATP Detection Assay

**Figure 3:**
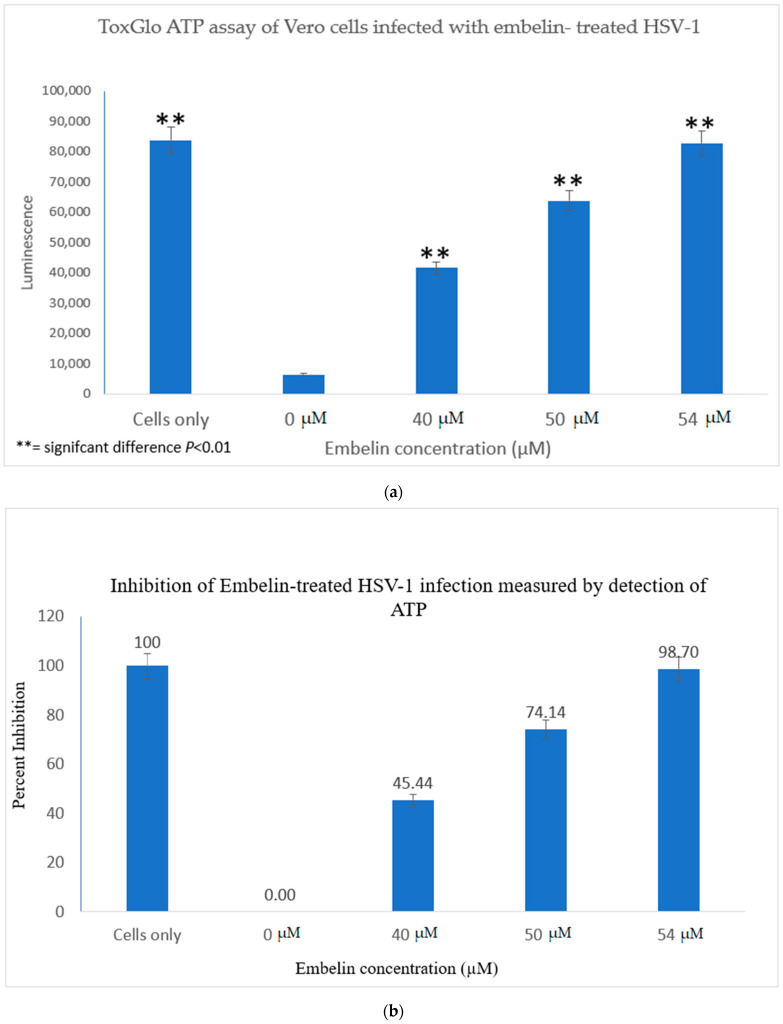
Treatment of HSV-1 with embelin affects cell viability. (**a**) The effect on cell viability of treatment of HSV-1 with various concentrations of embelin was determined by the Viral ToxGlo ATP assay. Viability was measured by luminescence (RLU) at 48 hpi. There was a significant difference in cell viability between cells infected with untreated virus and cells infected with HSV-1 treated with embelin at concentrations ranging from 40 to 54 µM; ** = significant difference *p* < 0.01. (**b**) The percent inhibition of HSV-1 infection following treatment of virions with embelin as measured by detection of ATP (indicating metabolically viable cells) reached 98.7% when HSV-1 was treated with 54 µM embelin.

The Viral ToxGlo assay quantifies cellular ATP as a measure of metabolically active viable cells This assay measured the effect of treatment of HSV-1 with various concentrations of embelin. Cell viability was measured by luminescence (RLU) at 48 hpi and is shown in Figure 3a.

The results indicated that the RLU decreased significantly for the 0 µM concentration (untreated HSV-1 control). The embelin treated HSV-1 increased the RLU in a dose dependent manner. The RLU of the 54 µM treated sample was equivalent to the RLU of uninfected Vero cells. This illustrated that 54 µM embelin treated HSV-1 does not affect cell viability (Figure 3a). The Student T test indicated that there are significant differences in cell viability when Vero cells are infected with HSV-1 treated with concentrations of embelin ranging from 40 to 54 µM as compared to the Vero cells infected with untreated HSV-1 (*p* < 0.01). 

The greatest percentage of inhibition was observed with the 54 μM treatment at over 98% inhibition followed by 74% inhibition at the 50 μM treated HSV-1. Percent inhibition, as measured by the ToxGlo ATP Detection assay is shown in Figure 3b. 

### 3.3. Microscopic Observation of Vero Cells, HSV-1 Infected Vero Cells and Embelin Treated HSV-1 Infected Vero Cells

#### 3.3.1. Inverted Microscopic Observation

**Figure 4:**
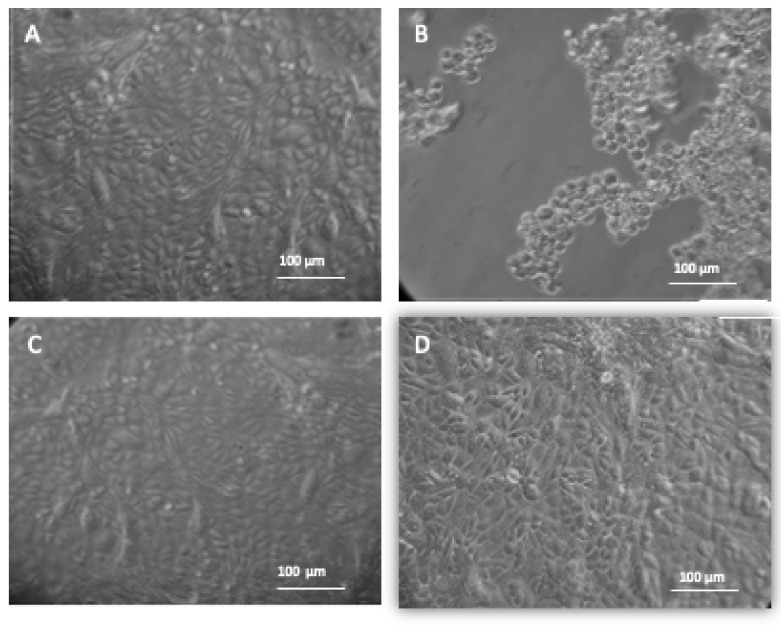
Treatment of HSV-1 with embelin protects Vero cells from cytotoxic damage. The morphology of Vero cells under different experimental conditions are shown. Images were taken through a Biostar inverted microscope (Reichert) following 48 h incubation at 37 °C and 5% CO_2._ (**A**) Vero cells; (**B**) Vero + HSV-1; (**C**) Vero cells + HSV-1 treated with 54 μM of Embelin; (**D**) Vero cells + 2% DMSO. Figure 4A shows Vero cells only and the morphology of the cells demonstrates a healthy, intact monolayer. Image 4B shows the effect of infecting Vero cells with HSV-1. Figure 4C shows no change in morphology of the Vero cells infected with HSV-1 treated with 54 μM of Embelin treated HSV-1 48 hpi. Figure 4D shows the administration of 2% DMSO presenting no toxicity for the vehicle solution.

To demonstrate the antiviral effects of embelin on HSV-1, the virus was treated with embelin for 1 h before infecting the Vero cells. Increasing concentrations of embelin were administered and 54 μM was confirmed to be the most effective at inhibiting the infection of HSV-1 in Vero cells (Figure 4C). Untreated HSV-1 (positive control) resulted in rounding and lifting of the Vero cell monolayer (Figure 4B). The morphology of the Vero cells infected with HSV-1 treated with embelin is consistent with the morphology of the uninfected Vero cells and the mock-infected treated with 2% DMSO (Figure 4D). The data demonstrate the antiviral effects of embelin at 54 µM concentration against HSV-1 (Figure 4C). This observation is consistent with the previous results of inhibition (Figure 2b and Figure 3b).

#### 3.3.2. Fluorescent Microscopy

**Figure 5:**
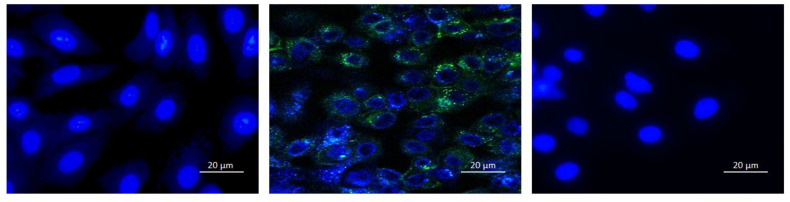
Expression of GFP is inhibited in HSV-1 infected cells when virions were treated with embelin. Fluorescent microscopy images of Vero cells only (**left**), HSV-1 infected cells (**middle**) and 54 μM Embelin treated HSV-1 infected cells (**right**) are shown. Viral particles with GFP appear green and the Vero cell nuclei are blue. Images were taken of the cells 12 hpi.

The HSV-1 strain contains a GFP fusion to the structural tegument protein pUL46. This enabled visualization of the effects of treatment of HSV-1 with embelin in infected cells through fluorescent microscopy 12 hpi (Figure 5). GFP (green) represents the virus and DAPI (blue) represents the nucleus of the host cells. There is a difference in the expression of GFP between the untreated and treated HSV-1 infected Vero cells at 12 hpi. 

### 3.4. Study of the Mechanisms of Embelin on HSV-1 Infective Cycle

#### 3.4.1. Inhibition of Binding by Embelin Treated HSV-1

**Figure 6:**
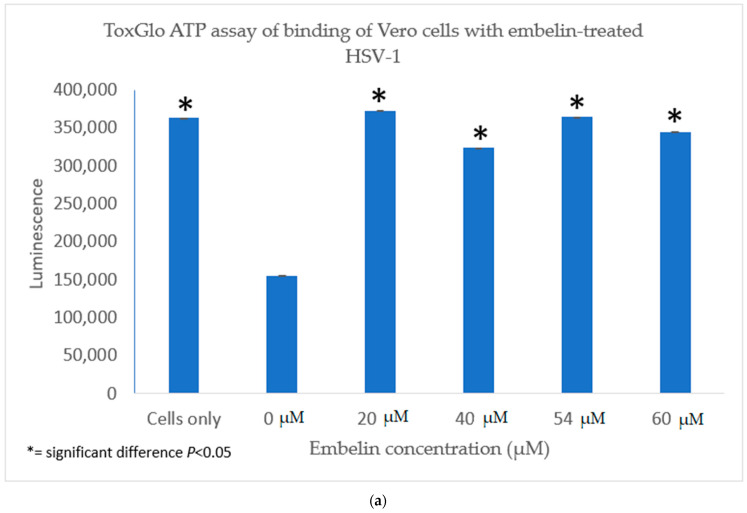

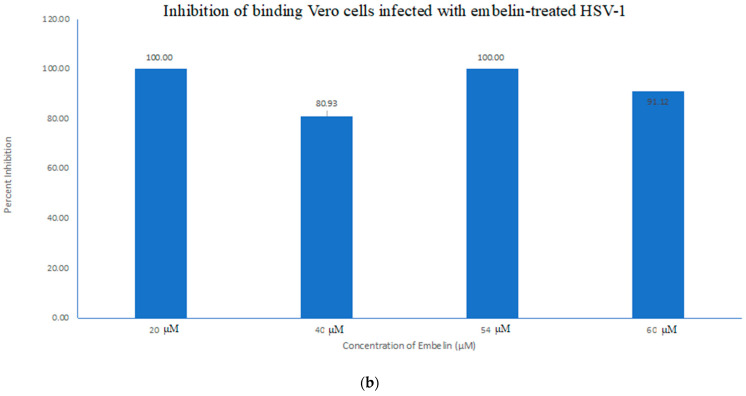
Treatment of HSV-1 virions with embelin inhibits binding. (**a**) The antiviral effects of embelin on the binding step of the viral cycle are reported. Cell viability was measured by luminescence of Vero cells following infection with different concentrations of embelin treated HSV-1. (**b**) The results of the percent inhibition resulting from treatment of HSV-1 with various concentrations of embelin ranging from 20 µM to 60 µM are reported.

The antiviral activity of embelin on the binding of HSV-1 virions to host cells was determined by infecting Vero cells with HSV-1 treated with increasing concentrations of embelin for 1 h at 4 °C. This allowed for the binding to occur but not the penetration of the virus. After 48 h, luminescence was measured. There was a significant difference between the binding of treated virions and untreated virions (Figure 6a). Inhibition of binding was significant when antiviral activity was calculated as a result of treatment of HSV-1 with concentrations of embelin as low as 20 µM (Figure 6b). This indicates that embelin inhibits the binding stage of the HSV-1 infection cycle.

#### 3.4.2. Inhibition of Penetration by Embelin Treated HSV-1

**Figure 7:**
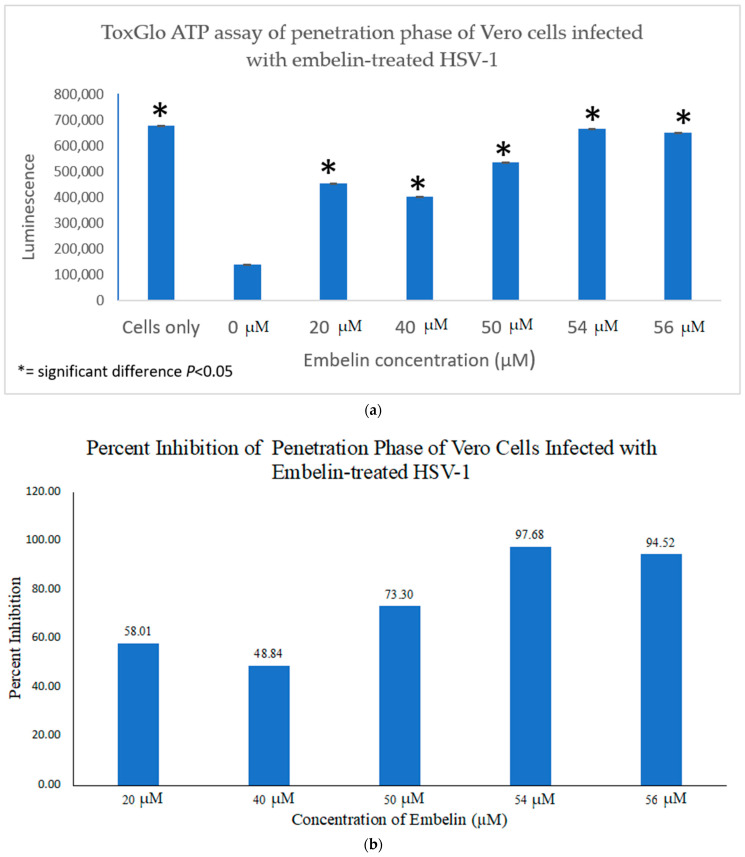
Treatment of HSV-1 with embelin inhibits the penetration step. (**a**) Cell viability as measured by luminescence 48 hpi indicates that treatment of virions with embelin significantly increased cell viability. (**b**) The percent inhibition of penetration stage of HSV-1 infection when virions were treated with embelin at concentrations ranging from 20 µM to 56 µM is reported. The greatest percentage of inhibition resulted from treatment of virions with 54 µM embelin.

In order to determine the antiviral effects of embelin at the point of penetration of the virus into the host cell, Vero cells grown in a 96 well plate were infected with HSV-1 at 4 °C for 2 h to ensure attachment but not penetration of the virions. Then a treatment of the cells for 10 min was performed at varying concentrations of embelin at room temperature during the penetration step [55]. After the addition of 100 μL Viral ToxGlo ATP Detection Buffer, luminescence was measured. Effects of treatment with embelin were observed (Figure 7a). Luminescence, which is correlated with cell viability, increased as embelin concentration increased. Highest cell viability occurred when HSV-1 was treated at 54 μM and 56 μM concentrations, respectively, with no significant difference determined between these and the Vero cell control. Treatment with each concentration, however, resulted in a significant difference between infection with the untreated HSV-1 (0 µM) and infection with each HSV-1 treated with each concentration of embelin. When the antiviral activity was calculated, 54 μM and 56 μM treatments had the highest percent inhibition reaching over 97% and 94%, respectively (Figure 7b). This demonstrates that embelin has antiviral activity in the penetration step of the viral cycle. The data indicated that treatment of HSV-1 with embelin affected antiviral activity during the initial stages of viral entry in the infection cycle—binding (Figure 6a,b) and penetration (Figure 7a,b).

#### 3.4.3. Inhibition of Post Penetration by Embelin Treated HSV-1

**Figure 8:**
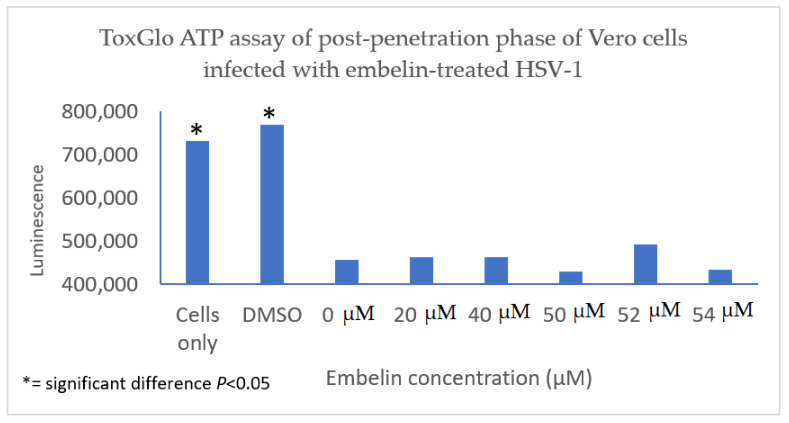
Treatment of HSV-1 infected cell with embelin post penetration does not affect cell viability. HSV-1 infected cells were treated with concentrations of embelin ranging from 20 µM to 54 µM post-penetration. DMSO represents the vehicle control up to 2% administration of DMSO. There is no significant difference in cell viability when HSV-1 infected cells are treated with embelin.

The antiviral effect of embelin on the post penetration step of the viral cycle was determined by infecting Vero cells with HSV-1 at 4 °C for 2 h to ensure attachment but not penetration of the HSV-1. Cells were then treated with embelin after the virus had entered the cells for 30 min at 37 °C and 5% CO_2_. Cell viability was measured 48 hpi. The results indicated that treatment with embelin after adsorption and penetration did not affect HSV-1 infection. There is minimal difference in the values obtained with addition of various concentrations of embelin and amount of luminescence resulting from viral infection control (0 µM embelin) (Figure 8). This further suggested that embelin is able to inhibit early stages of the virus infection cycle but has no effect after the entry of HSV-1. 

### 3.5. Effect of Antiviral Treatment on Circulating Redox Balance

**Figure 9:**
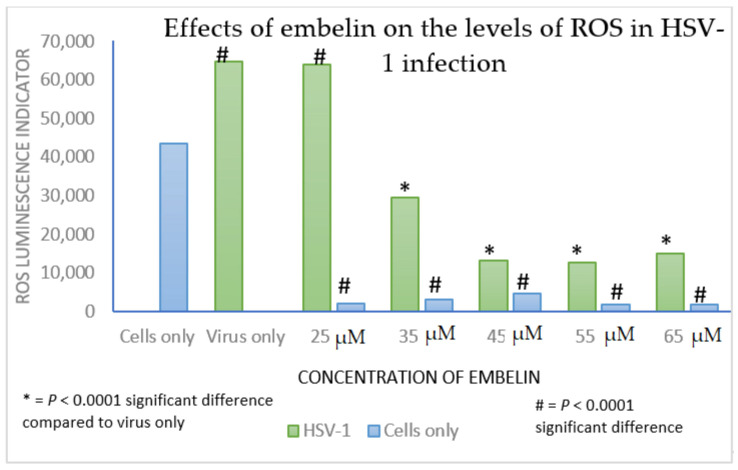
ROS generation is decreased in Vero cells treated with embelin at concentrations ranging from 25 to 65 µM and in Vero cells infected with embelin treated HSV-1 at concentrations of embelin ranging from 35 to 65 µM. Vero cells had lower overall H_2_O_2_ levels when infected with embelin treated HSV-1 in comparison to the untreated HSV-1 control. Statistical analysis was performed using the Excel function TTEST. The analysis used two tailed distribution with two-sample unequal variance test.

H_2_O_2_ induces oxidative stress in vascular cells [56]. To assess the impact of treatment with embelin on protection from oxidative damage, we measured production of H_2_O_2_ as an indicator of oxidative stress. Oxidative stress is determined by an extensive production of H_2_O_2_. When Vero cells were treated with embelin and an H_2_O_2_ substrate solution, there was a significant reduction in H_2_O_2_ as compared to production in untreated Vero cells at each concentration tested (25–65 µM embelin). Treatment of virions with concentrations of embelin ranging from 35 to 60 µM significantly reduced the production of the ROS H_2_O_2_ (Figure 9). These results indicate that embelin reduces oxidative damage to Vero cells and the oxidative damage caused by HSV-1 infection. 

## 4. Discussion

One strategy that viruses use to manipulate host cell machinery in viral infections is modulation of the intracellular redox state. An imbalance of the redox state towards oxidant conditions is a key event during viral infections. Polyphenols are antioxidants that may protect cells against oxidative damage. This study explored the role of the antioxidant embelin as an antiviral agent against HSV-1 infection of cultured Vero cells. Embelin was determined to be non-cytotoxic to Vero cells at the concentrations tested. Treatment of virions with embelin resulted in increased cell proliferation and viability, inhibition of virus infection at the early stages of infection, and reduction in the levels of hydrogen peroxide, H_2_O_2_. H_2_O_2_ is a major redox metabolite that functions in redox sensing, signaling and regulation [57]. It occurs as a metabolite of oxygen in aerobic metabolism in mammalian cells [58]. HSV-1 infection of neural cells increased ROS levels as early as 1 hpi and ROS levels remained elevated at 24 hpi [59].

Infection of Vero cells with HSV-1 led to increased ROS levels in addition to decreased ATP in cells. Embelin effectively inhibited the cytotoxic effects of HSV-1 infection. The inhibitory effect of embelin may be attributed to its role as an antioxidant.

In this study it was determined that embelin has no toxic effects on the cell up to 70 μM (Figure 1). This finding aligned with previous studies that report embelin as a safe, non-cytotoxic compound [41,42,52]. We then treated the HSV-1 virions with embelin to observe the antiviral effects. Cell proliferation, an indicator of the cellular response to embelin treatment on HSV-1, infection was measured. Treatment of HSV-1 with embelin at concentrations ranging from 40–54 µM effectively inhibited infection. Treating the virus resulted in 102.5% cell viability as compared to uninfected cells (Figure 2a). When the percent inhibition was calculated, the percent inhibition was the highest at 54 μM calculated at 100% and 98.7%, respectively (Figure 2b and Figure 3b). Cell viability was also assessed by measuring the amount of ATP in infected cells (Figure 3a). The percent of inhibition, as determined by the detection of ATP, reached 98.7% when HSV-1 was treated with 54 µM concentration of embelin. Antiviral effects of embelin were observed using microscopy at an inhibitory concentration of 54 µM (Figure 4 and Figure 5). The antiviral effect of embelin was also demonstrated by the reduction in the synthesis of HSV-1 DNA (Figure 3).

We investigated the inhibitory mechanism of embelin. Treatment with embelin affected the early stage of HSV-1 infection of Vero cells, the efficacy of which may be due to the insertion of embelin in the Vero cell membrane [41]. Embelin, at concentrations ranging from 20 to 60 µM, inhibited the binding stage of HSV-1 infection (Figure 7a,b) and penetration at concentrations ranging from 20 to 56 µM (Figure 7a,b). The proposed antiviral mechanism of embelin is consistent with the action of other antivirals [23,25,26,27,28]. Additionally, this action may be attributed to the excellent antioxidant activity of embelin, when sequestering the superoxide radical [40,41]. 

Computational studies have revealed the mechanism by which quinone derivatives can inhibit SARS-CoV-2 {53]. Previous studies reported specific modes of action in the biological activities of embelin. Embelin inhibited the nuclear factor—κB (NF-κB) signaling pathway leading to suppression of NF-κB anti-apoptotic and metastatic gene products [60]. Embelin suppressed paraquat-induced lung injury through suppressing oxidative stress, inflammatory cascade, and MAPK/NF- κB signaling pathway [61]. As an anti-cancer agent, embelin suppressed the signal transducer and activator of transcription 3 (STAT-3) pathway to suppress cell proliferation and invasion in cancer cells [62]. 

Viral infections result in oxidative cell damage due to disruption of cellular antioxidant mechanisms [63]. ROS levels increased in neural cells following HSV-1 entry and replication [59]. HSV-1 infection and oxidative stress have been linked to the neurodegeneration associated with Alzheimer’s disease [64]. Influenza A infection of cultured cells led to increased levels of ROS that correlated with an increase in virus titer [32]. Additionally, oxidative stress is involved in cellular damage associated with hepatitis C and human immunodeficiency co-infections and suggest that altered redox status promotes cellular damage [65]. Direct acting antivirals restored circulating redox homeostasis in patients affected with chronic hepatitis C infection [39]. Curcumin was found to be an inhibitor to APE1 (Apurinic/apyrimidinic endonuclease1) redox function, affecting many genes and pathways. Curcumin inhibited Kaposi’s sarcoma-associated herpesvirus (KSHV) lytic replication [38]. We have shown that treatment with embelin reduced ROS caused by HSV-1 infection (Figure 9). Future studies are required to provide additional information on the therapeutic potential of embelin, such as determining the selectivity index required for drug development.

## 5. Conclusions

In conclusion, embelin reduces oxidative damage caused by HSV-1 infection and is an effective antiviral to reduce the infection of HSV-1 in cultured Vero cells. Further studies are needed to explore the possibility of embelin as a medicinal agent.

## Data Availability

The data presented in this study are available in the text of this article.
